# Novel Duck Orthoreovirus Induces Ferroptosis in HD11 Cells by Hijacking Cellular Iron Metabolism and Promoting Iron Accumulation

**DOI:** 10.1155/tbed/7722201

**Published:** 2026-01-16

**Authors:** Hongzhi Wang, Di Lei, Chenchen Jiang, Boyi Xu, Yi Tang, Rendong Fang

**Affiliations:** ^1^ College of Veterinary Medicine, Southwest University, Beibei District, Chongqing, China, swu.edu.cn; ^2^ Institute of Animal Sciences of Chinese Academy of Agricultural Sciences, Haidian District, Beijing, China, caas.cn

**Keywords:** ferroptosis, iron metabolism, macrophage, novel duck orthoreovirus

## Abstract

Novel duck orthoreovirus (NDRV) infection induces severe splenic necrosis in ducks, resulting in a cascade of detrimental consequences, including immunosuppression, secondary infections, and diminished vaccine efficacy. Avian orthoreovirus (ARV) exhibits high tropism for macrophages, with splenic macrophages being identified as the primary target cells of NDRV. Although ferroptosis has been implicated in this pathological process, the molecular mechanism underlying NDRV‐induced cellular damage remains poorly elucidated. In this study, an in vitro model of NDRV infection was established using HD11 cells to systematically investigate its effect on ferroptosis and the associated mechanisms. Our results indicate that NDRV infection triggers ferroptosis and markedly elevates intracellular Fe^2+^ levels. Mechanistically, NDRV upregulates transferrin receptor 1 (TfR1), thereby enhancing iron uptake, promoting iron accumulation, and ultimately inducing ferroptosis. This study is the first to reveal that NDRV induces macrophage ferroptosis by hijacking cellular iron metabolism, providing a theoretical foundation for understanding the mechanism through which NDRV infection mediates splenic necrosis and immune cell injury.

## 1. Introduction

In recent years, avian orthoreovirus (ARV) has evolved through point mutations, genetic recombination, and cross‐species transmission, posing an increasingly significant threat to the poultry industry in China [1]. Since 2017, duck splenic necrosis disease caused by novel duck orthoreovirus (NDRV) has been extensively reported across the country. This disease is primarily characterized by severe splenic necrosis, which can lead to immunosuppression, predispose hosts to secondary infections with various pathogens, and substantially undermine vaccine effectiveness, with mortality rate reaching up to 50%, resulting in considerable economic losses [2]. However, the molecular mechanisms underlying NDRV‐induced splenic necrosis remain poorly understood.

Virus‐induced cellular damage represents the initial event in the pathogenesis of tissue injury. Research on ARV‐induced host cell injury began in 1989 [3]. ARV has been shown to trigger apoptosis in various avian and some mammalian cells, such as DF‐1 cells, through interaction with the host eukaryotic translation elongation factor 1α1 (EEF1A1) [4]. In Vero cells, apoptosis occurs via p53‐dependent pathways or activation of transcription factor 6 (ATF6) [5], whereas autophagy in CEF and Vero cells is mediated by the PI3K/Akt/mTOR axis [6]. However, despite the fact that numerous studies have been dedicated to uncovering the interaction mechanisms between ARV and pattern cells, the specific pathways by which the virus causes damage to host target cells remain poorly understood.

In a previous duck model of NDRV infection, the pathological progression of splenic necrosis was systematically investigated [7]. During the early stage of infection (Day 2), splenic swelling and scattered necrotic foci were observed. As the disease progressed (Days 5–7), lesions worsened, marked by extensive necrosis and tissue sclerosis, with severe pathology persisting up to 15 days postinfection. Histopathological analysis revealed dynamic changes in the splenic parenchyma, including congestion, lymphocyte depletion, hemosiderin deposition, macrophage infiltration, and granuloma formation—findings consistent with those reported by Kong et al. [7, 8]. While these studies clarify the pathology of NDRV‐induced splenic necrosis, the underlying mechanisms governing splenic cellular damage remain to be fully elucidated.

Ferroptosis is an iron‐dependent form of programmed cell death characterized by the accumulation of lipid peroxides [9]. It is critically involved in the onset and progression of various diseases and has been widely implicated in the pathophysiological processes associated with pathogenic infections [10, 11]. Accumulating evidence indicates that viruses can modulate ferroptosis to facilitate their replication and exacerbate host tissue damage [12, 13]. For instance, Newcastle disease virus (NDV) triggers ferroptosis in human glioma cells via the p53‐SLC7A11‐GPx4 signaling axis to promote viral replication [14]. H5N1 avian influenza virus (IV) upregulates tripartite motif‐containing protein 21 (TRIM21) expression, inducing oxidative stress and ferroptosis through the SQSTM1‐Nrf2‐KEAP1 pathway to facilitate viral proliferation [15]. Furthermore, ferroptosis contributes to mechanisms of viral immune evasion. Influenza A virus (IAV), for example, induces ferritinophagy via hemagglutinin, resulting in lipid peroxidation and suppression of mitochondrial antiviral signaling protein (MAVS) aggregation, ultimately impairing MAVS‐mediated antiviral immunity [16]. Our previous RNA‐seq data indicate that ferroptosis represents a predominant mode of host cell death during NDRV infection [17], yet its regulatory mechanisms in the context of NDRV infection remain unclear.

Macrophages, as central regulators of iron metabolism, play a central role in maintaining systemic iron homeostasis [18]. Iron overload can trigger ferroptosis in macrophages and exacerbate tissue injury. For instance, the ferroptosis inhibitor Ferrostatin‐1 (Fer‐1) has been shown to significantly attenuate liver damage in experimental models of iron overload [19]. Similarly, in rheumatoid arthritis (RA), excessive iron accumulation in synovial fluid correlates with disease severity and promotes RA progression by inducing macrophage ferroptosis [20]. These findings underscore the critical involvement of macrophage ferroptosis in various pathological conditions. Furthermore, given that macrophages perform critical immune functions—including migration, phagocytosis, and antigen presentation—studying how pathogens impair these cells is essential for understanding microbial pathogenesis and mechanisms of immune evasion.

Elucidating whether and how NDRV induces macrophage ferroptosis is crucial for understanding the molecular mechanisms of NDRV‐induced splenic necrosis. This study investigates the interplay between NDRV and host iron metabolism from the emerging perspective of ferroptosis, which may provide novel insights into NDRV pathogenesis and aid the development of ferroptosis‐targeted antiviral strategies.

## 2. Materials and Methods

### 2.1. Virus, Cell Lines, and Antibodies (Abs)

The NDRV strain employed in this study was isolated from a deceased duckling in Shandong Province, China. LMH and HD11 cells were cultured in DMEM/F12 medium (Procell, PM150312, China) supplemented with 10% fetal bovine serum (FBS; ExCell Biotech, FSP500, China), 100 U/mL penicillin, and 100 μg/mL streptomycin (Sangon Biotech, E607011, China). All cell cultures were maintained under standard conditions at 37°C with 5% CO_2_. Polyclonal Abs against NDRV σA protein were generated in our laboratory by immunizing rabbits with recombinant σA protein. Abs against TfR1 was purchased from Affinity Biosciences (Catalog Number AF5343, China). Abs against PTGS2, GPx4, FTH1, and ferroportin (Fpn) were purchased from Abcam (Catalog Numbers ab179800, ab125066, ab75973, and ab239511, Britain). Abs against α‐tubulin was purchased from Proteintech (Catalog Number 10144, USA).

### 2.2. RNA Extraction and Quantitative PCR

Total RNA was extracted using TRIzon Total RNA Extraction Reagent (Cwbio, CW0580S, China), and cDNA was synthesized using a reverse transcription kit (Cwbio, CW2569M, China). Quantitative real‐time PCR (qPCR) was performed on the LightCycler 96 system (Roche, LC96, USA) using gene‐specific primers designed using Primer 5 software. The sequences of the primers and probes used in qPCR are listed in Table 1. Relative gene expression levels were calculated using the 2^-ΔΔCT^ method, with β‐actin serving as the reference gene.

**Table 1:** Primers and probe used in this study.

Primer name	Sequence (5’–3’)	
	Forward/sense	Reverse/anti‐sense
NDRV‐S2	CCCGGATTCTCGATGAATGGT	CGACCCACTGCTGGATACAAG
NDRV‐S2‐Probe	FAM‐5’r‐AACGCCTGTGCACGAGCTGAAC‐3’‐TAMRA r	
TfR1	CTCCTTTGAGGCTGGTGAGG	CACTTGGTTCTTGGTGCTGC
FTH1	GTACTTCCTGCACCAGTCCC	CTCCCAGTCATCACGATCCG
Fpn	TTACCTTGGACATGCGCTGT	CCTCGAGTTCTTGTCCACCC
PTGS2	TCCACCGGTAGGACATGACT	GCCAGGCCCTTTCTTATGGT
GPx4	AAATGAGGAAAGACCGCGGT	CATGTCGAACTTCACCCCGT
β‐Actin	TGATATTGCTGCGCTCGTTG	AACCATCACACCCTGATGTCTG
siRNA‐NC	UUCUCCGAACGUGUCACGUTT	ACGUGACACGUUCGGAGAATT
siRNA‐TfR1	GGCACUGGAACUGCUAUAUTT	AUAUAGCAGUUCCAGUGCCTT

### 2.3. Indirect Immunofluorescence Assay (IFA)

At 24 h postinfection, cells were fixed with 4% paraformaldehyde (Beyotime, P0099, China) for 15 min, permeabilized with 0.2% Triton X‐100 (Beyotime, P0096, China) for 10 min, and blocked with 5% bovine serum albumin (BSA; Beyotime, ST2249, China) for 1 h at room temperature (RT). Cells were incubated overnight at 4°C with rabbit anti‐NDRV σA polyclonal antibody, washed, and then incubated with FITC‐conjugated goat anti‐rabbit IgG (1:300 dilution; Transgen, HS111, Germany) for 1 h at RT. Nuclei were stained with Hoechst 33,342 (Beyotime, C1026, China) for 5 min at RT. Cellular images were captured using a fluorescence microscope.

### 2.4. Western Blot Analysis

HD11 cells were either subjected to NDRV infection following established protocols or transfected with siRNA using Lipo8000 transfection reagent (Beyotime, C0053, China). The treated cells were lysed with IP lysis buffer (Biosharp, BL509, China), and lysates were centrifuged to collect the supernatants. Protein concentrations were determined using the BCA assay. SDS‐PAGE loading buffer was added, and samples were heated at 95°C for 15 min. Proteins were separated via 10% SDS‐PAGE gels and transferred onto PVDF membranes (0.22 μm) at 180 mA for 1.5 h. Membranes were blocked with 5% nonfat milk or BSA prepared in TBST for 1 h at RT, then incubated with primary Abs overnight at 4°C. After three washes with TBST, membranes were incubated with HRP‐conjugated secondary antibody (1:6000, Cwbio, CW0102S/0103S, China) for 1 h at RT. Following subsequent washes, protein bands were visualized using an ECL chemiluminescence kit (Biosharp, BL523B, China).

### 2.5. Cell Viability Assay

Cell viability was assessed using the WST‐1 Cell Proliferation and Cytotoxicity Assay Kit (Beyotime, C0035, China). Briefly, 10 μL of WST‐1 solution was added to each well containing treated cells, followed by incubation at 37°C in the dark for 2 h. The reaction mixture was subsequently gently shaken to ensure homogeneity. Absorbance at 450 nm was measured using a microplate reader, and cell viability was determined in accordance with the manufacturer’s protocol.

### 2.6. Ultrastructural Observation of Cellular Components

Following a wash with PBS, infected cells were fixed with 2.5% glutaraldehyde for 5 min at RT. Cells were then gently scraped unidirectionally with a cell scraper, transferred to a 2 mL microcentrifuge tube, and centrifuged at 2000 rpm for 2 min. The supernatant was carefully removed, and the cell pellet was resuspended in fresh 2.5% glutaraldehyde fixative. Postfixation was carried out at RT in the dark for 30 min, after which samples were stored at 4°C for 24 h. Subsequently, cells were dehydrated through a graded acetone series (50%, 70%, 80%, 90%, and 100%), embedded in epoxy resin, and polymerized for ultrathin sectioning. Ultrathin sections were stained with uranyl acetate for 10 min and counterstained with lead citrate for 5 min. Cellular ultrastructure was examined using transmission electron microscopy, and representative micrographs were acquired.

### 2.7. Assessment of Intracellular Labile Iron Levels

Intracellular Fe^2+^ levels were determined using the FerroOrange fluorescent probe (Dojindo Laboratories, F374, Japan) according to the manufacturer’s instructions. After washing with PBS, treated cells were incubated with 1 μmol/L FerroOrange working solution at 37°C with 5% CO_2_ in the dark for 30 min. The staining solution was then removed and cells were washed three times with PBS. Subsequently, Hoechst 33342 staining solution (Beyotime, C1017, China) was added to label cell nuclei, and incubation at RT for 5 min. After an additional PBS wash, coverslips were mounted with an antifade mounting medium and sealed using neutral balsam. Fluorescence images were acquired via a laser scanning confocal microscope.

### 2.8. Detection of Lipid ROS

The intracellular lipid ROS were quantified using the highly sensitive DCFH‐DA fluorescent probe (Dojindo Laboratories, R252, Japan) following the manufacturer’s instructions. Phorbol 12‐myristate 13‐acetate (PMA, Beyotime, S1819, China), a widely used ROS activator, was employed as a positive control treatment in this study. Briefly, after washing the treated cells with basal medium, 100 μL of DCFH‐DA working solution was added per well and incubated at 37°C for 20 min. The DCFH‐DA solution was then carefully removed, and the cells were washed three times with basal medium to eliminate any unincorporated probe. Thereafter, Hoechst 33342 staining solution was added to label the nuclei, followed by incubation at RT for 5 min. After a final wash with PBS, cells were examined under a fluorescence microscope, and representative images were captured.

### 2.9. Measurement of Malondialdehyde (MDA)

To assess intracellular lipid peroxidation, MDA levels in treated cells were measured using a lipid peroxidation assay kit (Beyotime, S0131S, China) following the manufacturer’s instructions. Cells were harvested and lysed as directed. For each 100 μL sample, 200 μL of MDA detection working solution was added, thoroughly mixed, and incubated in a boiling water bath for 15 min. After cooling to RT, 200 μL of supernatant was transferred to a 96‐well plate, and absorbance was measured at 532 nm. The molar concentration of MDA in each sample was subsequently determined based on a standard calibration curve.

### 2.10. Statistical Analysis

The average fluorescence intensity of regions with positive signals was quantified using ImageJ software by applying a consistent threshold. All data are presented as mean ± standard deviation unless otherwise specified. Intergroup statistical comparisons were conducted using GraphPad Prism software, with a *p*‐value <0.05 considered statistically significant. Significance levels are indicated as follows:  ^∗^ *p* < 0.05,  ^∗∗^ *p* < 0.01,  ^∗∗∗^ *p* < 0.001.

## 3. Results

### 3.1. Establishment of an NDRV Infection Model in HD11 Cells

Owing to the short culture cycle and low transfection efficiency of duck primary macrophages in vitro, the HD11 cell line—widely utilized in studies of avian pathogen infections—was employed to establish an in vitro NDRV infection model [21, 22]. Viral infection and replication were assessed using IFA and qPCR assay. The results showed that NDRV infection induced typical cytopathic effects (CPEs) in LMH cells, including shrinkage, aggregation into clusters, and ultimately detachment and floating (Figure 1A). In HD11 cells, when the MOIs was set at 1 and 5, evident CPE could be detected at 24 h and 48 h postinfection, primarily characterized by cell shrinkage and detachment (Figure 1B). qPCR analysis revealed a time‐dependent increase in the mRNA levels of the NDRV σA gene (Figure 1C). Furthermore, IFA detected specific fluorescent signals in infected HD11 cells (Figure 1D), suggesting active viral replication. These findings collectively demonstrate that NDRV can stably replicate in HD11 cells, and the established in vitro model is suitable for downstream experimental investigations.

Figure 1Construction of an HD11 cell model for NDRV infection. (A) LMH cells were infected with NDRV at an MOI of 5 for 24 and 48 h, and cytopathic effects were observed. (B) HD11 cells were infected under the same conditions, and cytopathic effects were also evident. (C) Total RNA was extracted from infected HD11 cells at 24, 48, and 72 h postinfection for qPCR. Viral copy number increased progressively over time. All experiments were performed in triplicate. (D) LMH and HD11 cells were infected at an MOI of 5, and viral protein expression was detected at 24 h postinfection using an anti‐σA polyclonal antibody.

**Figure:**
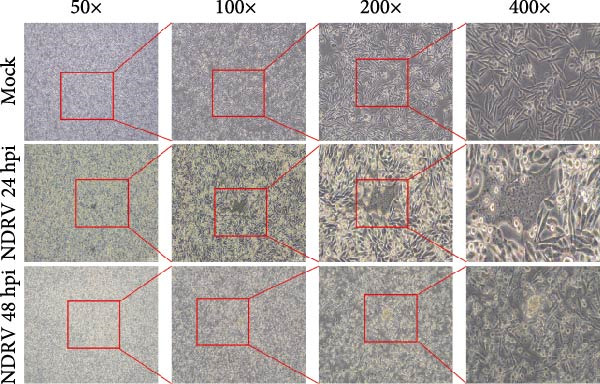
(A)

**Figure:**
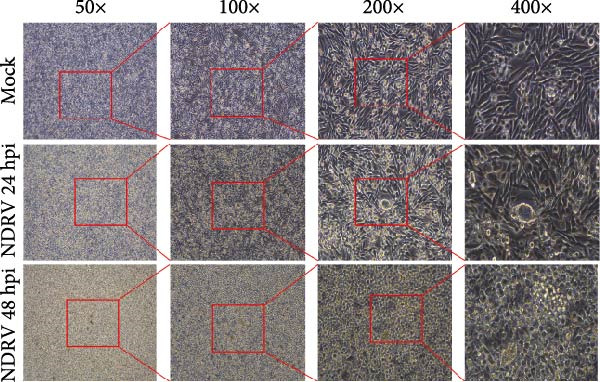
(B)

**Figure:**
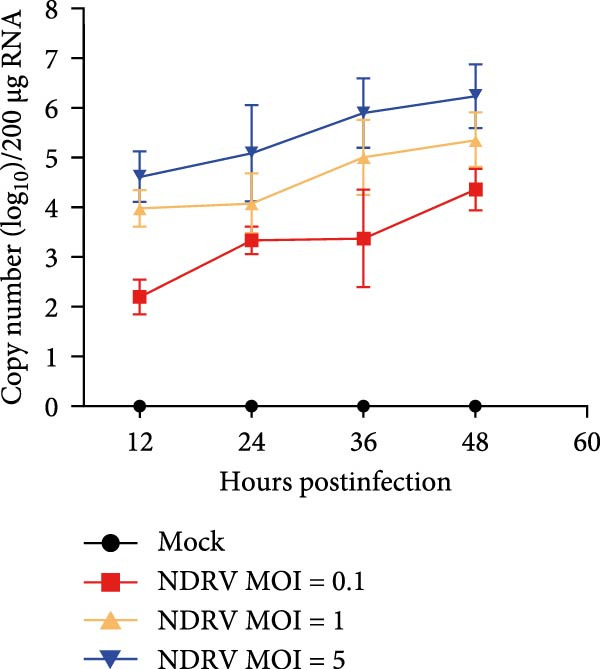
(C)

**Figure:**
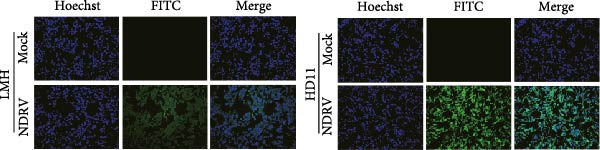
(D)

### 3.2. NDRV Induces Ferroptosis in HD11 Cells

To investigate whether NDRV induces ferroptosis in HD11 cells in vitro, cellular morphology and associated biological responses were examined postinfection. Transmission electron microscopy revealed mitochondrial changes characteristic of ferroptosis, including cristae fragmentation and reduced cristae density (Figure 2A). Western blotting and qPCR analyses further demonstrated significant changes in the expression levels of the ferroptosis‐related markers PTGS2 and GPx4 at both transcriptional and protein levels (Figure 2B,C). Intracellular ROS levels were evaluated using the DCFH‐DA fluorescent probe, which revealed increased ROS accumulation at 24 h postinfection, with a significant elevation observed at 48 h; in contrast, no notable change was detected in the control group (Figure 2D,E).

Figure 2NDRV induces ferroptosis in HD11 cells. (A) HD11 cells were infected with NDRV (MOI = 5) and fixed at 24 h postinfection. Transmission electron microscopy showed cristae fragmentation and reduced cristae density in the infected cells. At least 10 cells were examined under each condition. (B) Following NDRV infection, total protein was extracted from HD11 cells, and the expression levels of PTGS2 and GPx4 were analyzed by western blot. (C) Total RNA was isolated from infected cells, and PTGS2 and GPx4 mRNA levels were quantified by qPCR. (D–E) HD11 cells were infected with NDRV at MOIs of 1 and 5, and intracellular ROS levels were measured using DCFH‐DA at 24 h postinfection. NDRV increased ROS levels in HD11 cells. (F) ImageJ software was used to quantitatively analyze the fluorescence area, showing that intracellular ROS levels increased in a dose‐ and time‐dependent manner during NDRV infection, indicating a positive correlation. Significance levels are indicated as follows:  ^∗^ *p* < 0.05,  ^∗∗^ *p* < 0.01.

**Figure:**
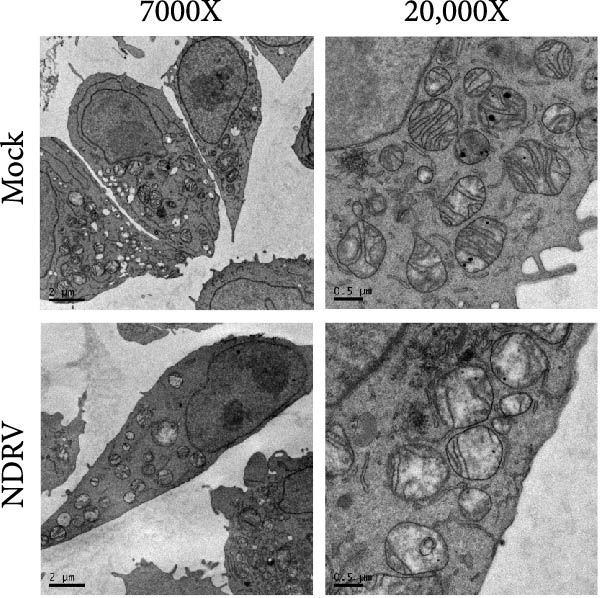
(A)

**Figure:**
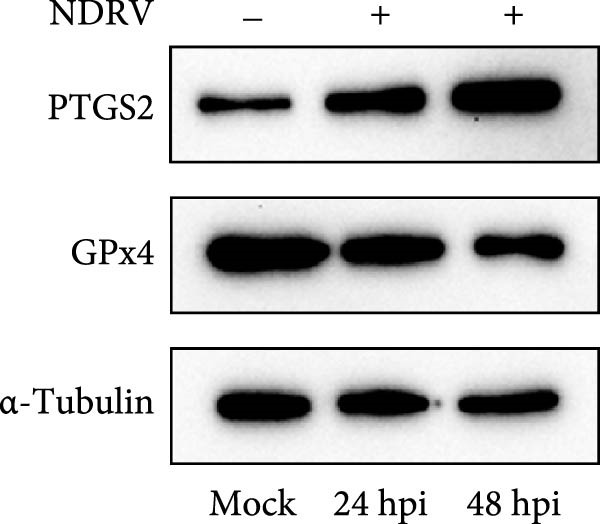
(B)

**Figure:**
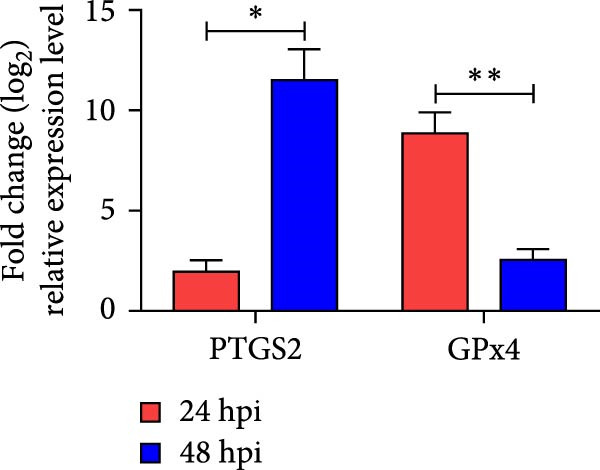
(C)

**Figure:**
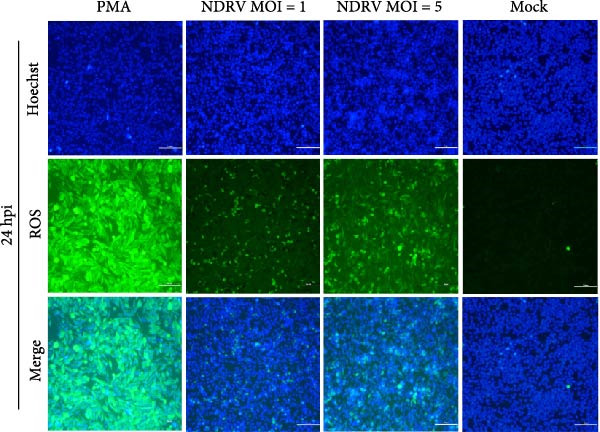
(D)

**Figure:**
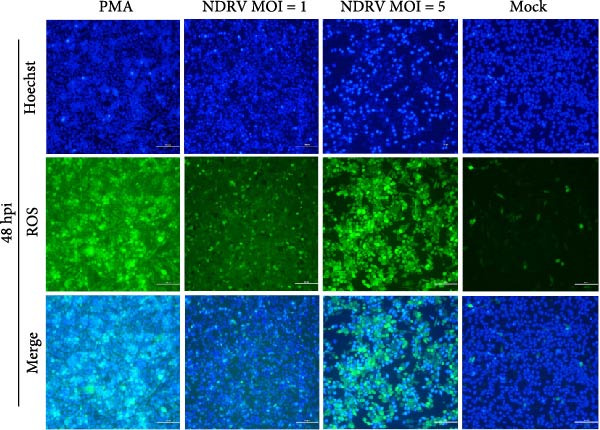
(E)

**Figure:**
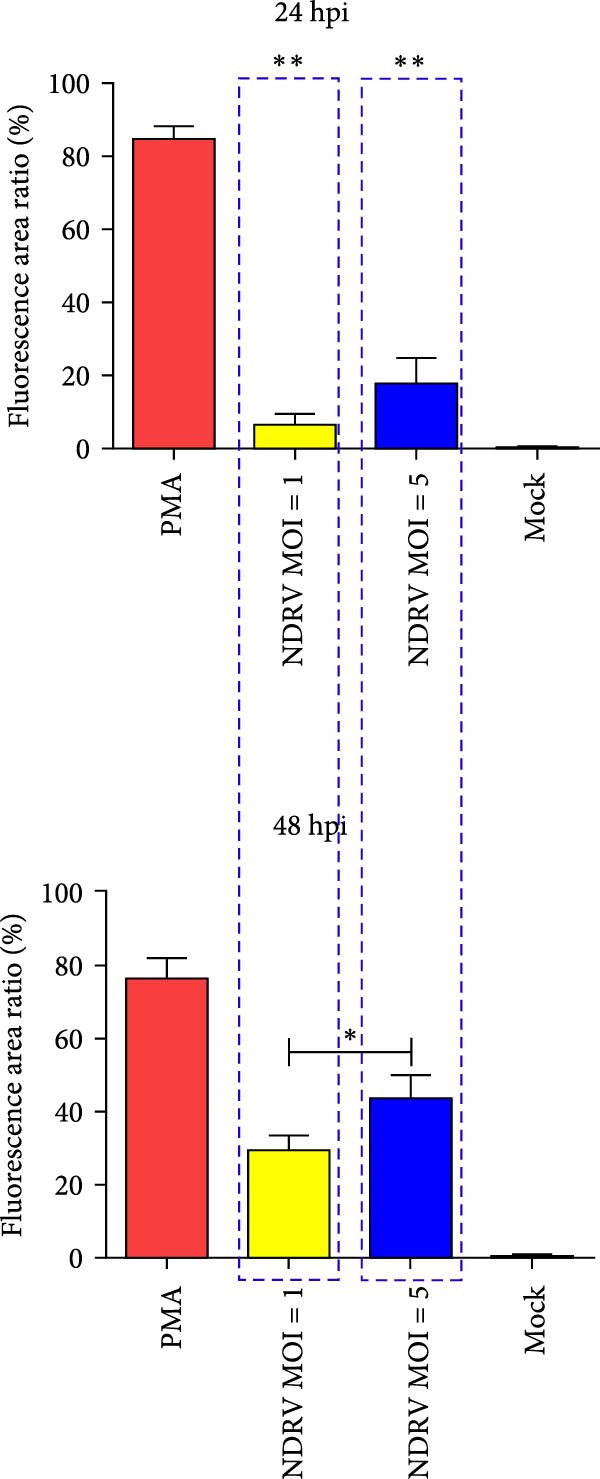
(F)

Quantification of the fluorescence area ratio using ImageJ software demonstrated that ROS accumulation was dose‐ and time‐dependent (Figure 2F). Collectively, these findings indicate that NDRV effectively induces ferroptosis in HD11 cells under in vitro conditions.

### 3.3. NDRV Infection Facilitates Fe^2+^ Accumulation in HD11 Cells

Macrophages play a critical role in the homeostasis of iron metabolism. Under physiological conditions, cells store iron in ferritin, export it via Fpn, or utilize it in biosynthetic pathways. However, the accumulation of intracellular labile iron ions, particularly Fe^2+^, can catalyze the Fenton reaction, leading to excessive production of ROS, which subsequently induces pronounced lipid peroxidation and ultimately triggers ferroptosis. In our previous study, we reported that NDRV infection significantly upregulated the expression of iron metabolism‐related genes in splenic cells. To investigate the impact of NDRV infection on the intracellular Fe^2+^ concentration in HD11 cells, changes in Fe^2+^ levels were monitored using the FerroOrange fluorescent probe.

The results revealed that Fe^2+^ content in HD11 cells increased significantly at 12 h postinfection (Figure 3A). This elevation became more pronounced at 24 h postinfection (Figure 3B), and the extent of accumulation was positively correlated with both viral dose and infection duration (Figure 3C).

Figure 3NDRV infection promoted Fe^2+^ accumulation in HD11 cells. (A) HD11 cells were infected with NDRV (MOI = 1, 5) for 12 h, then fixed and stained with FerroOrange to detect intracellular Fe^2+^ levels. (B) Intracellular Fe^2+^ levels were assessed using FerroOrange probe at 24 h postinfection. (C) Quantitative analysis of fluorescence signal area using ImageJ software demonstrated that Fe^2+^ levels increased in a dose‐ and time‐dependent manner following NDRV infection. Significance levels are indicated as follows:  ^∗^ *p* < 0.05.

**Figure:**
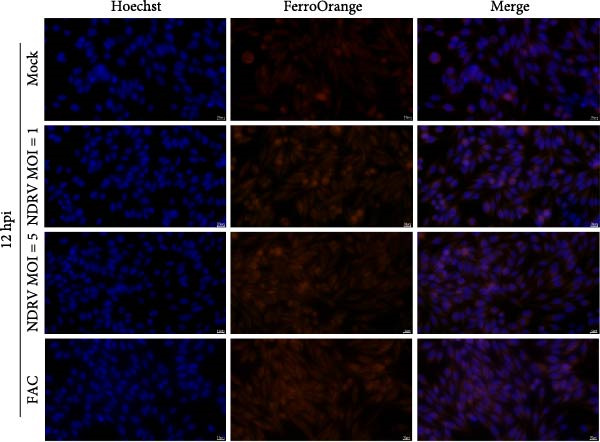
(A)

**Figure:**
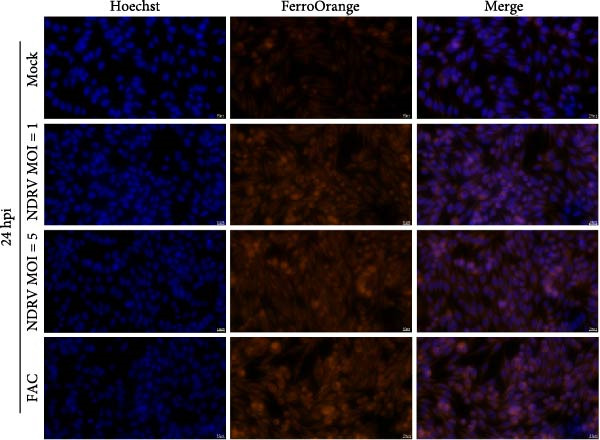
(B)

**Figure:**
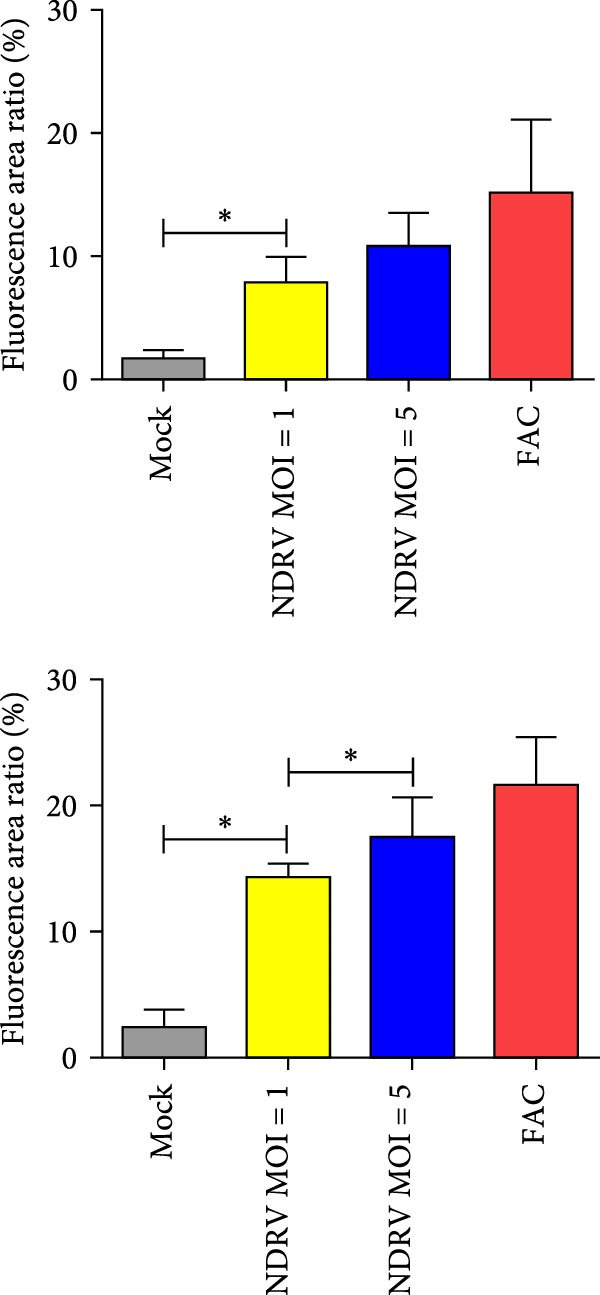
(C)

### 3.4. NDRV Induces Ferroptosis in HD11 Cells by Promoting Fe^2+^ Accumulation

To investigate the role of Fe^2+^ accumulation in NDRV‐induced ferroptosis in macrophages, HD11 cells were pretreated with the iron chelator deferoxamine (DFO) to reduce intracellular iron levels and ferroptosis sensitivity. DFO treatment alone did not significantly affect HD11 cell viability (Figure 4A), indicating that it was noncytotoxic at this concentration and suitable for further experiments. In the NDRV infection model, DFO pretreatment markedly alleviated the virus‐induced reduction in cell viability (Figure 4B), suggesting a protective role against viral CPEs. Further analysis of ferroptosis‐related biochemical markers showed that DFO significantly reduced the accumulation of MDA, a product of lipid peroxidation, in NDRV‐infected HD11 cells (Figure 4C), and effectively suppressed the elevation of ROS levels (Figure 4D). These findings indicate that DFO alleviates NDRV‐induced oxidative stress and lipid peroxidation through iron chelation, thereby inhibiting ferroptosis. Taken together, these results demonstrate that Fe^2+^ accumulation is a key mechanism triggering ferroptosis in NDRV‐infected HD11 cells.

Figure 4NDRV induces ferroptosis in HD11 cells by promoting Fe^2+^ accumulation. (A) HD11 cells were treated with the iron chelator deferoxamine (DFO; 100 μM), and viability was assessed to evaluate DFO’s effect. (B) HD11 cells were infected with NDRV and concurrently treated with or without DFO to assess DFO’s ability to mitigate NDRV‐induced cytotoxicity. (C) Lipid peroxidation levels in NDRV‐infected HD11 cells following DFO treatment were measured using an MDA assay kit. (D) ROS levels in NDRV‐infected HD11 cells, with or without DFO treatment, were assessed using the DCFH‐DA fluorescent probe. Significance levels are indicated as follows:  ^∗^ *p* < 0.05,  ^∗∗^ *p* < 0.01.

**Figure:**
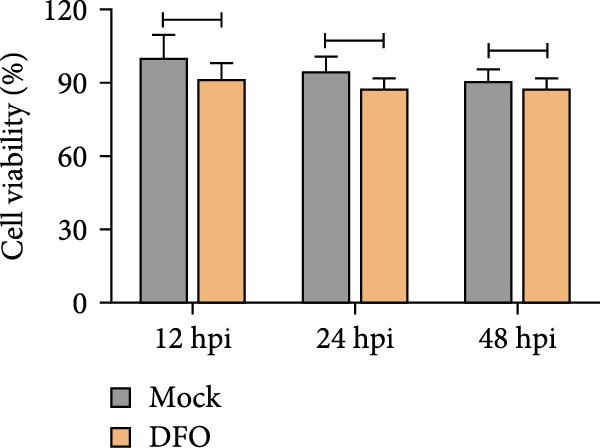
(A)

**Figure:**
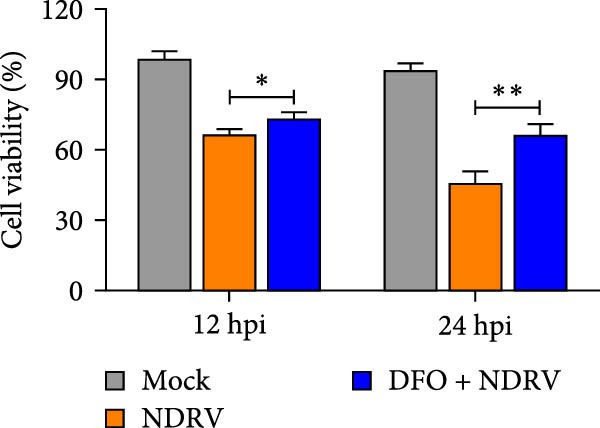
(B)

**Figure:**
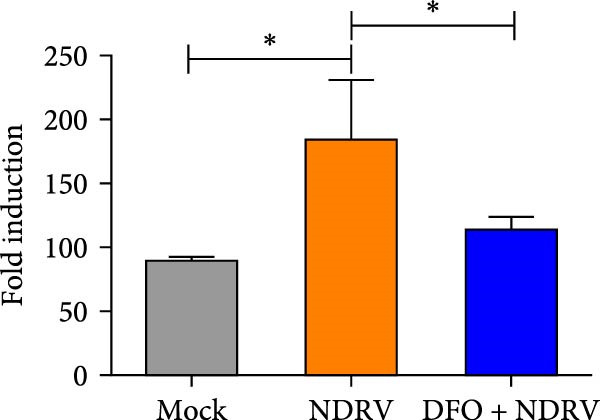
(C)

**Figure:**
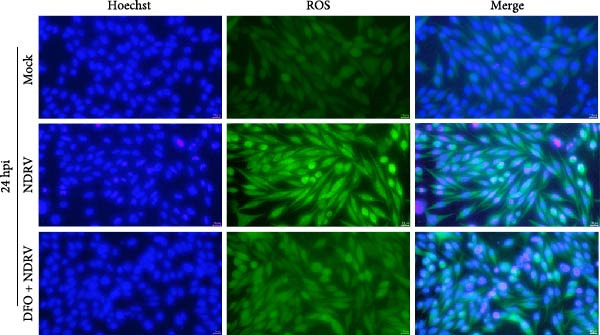
(D)

### 3.5. NDRV Triggers Ferroptosis in HD11 Cells by Upregulating TfR1 Expression

NDRV infection upregulates iron metabolism‐related genes (e.g., TF, TfR1, Hmox1, ACO1, and STEAP3) and downregulates the iron export gene Fpn. As a key membrane receptor mediating cellular iron uptake, TfR1 plays a crucial regulatory role in ferroptosis. Our results demonstrate that NDRV infection significantly enhances TfR1 expression at both the mRNA and protein levels in HD11 cells (Figure 5A,B). To further investigate the functional role of TfR1 in virus‐induced ferroptosis, we employed RNA interference to knockdown TfR1 expression, which markedly reduced the NDRV‐induced increase in intracellular Fe^2+^ concentration (Figure 5C), indicating that TfR1‐mediated iron uptake is a central mechanism underlying iron accumulation during NDRV infection. Furthermore, viral infection led to a significant rise in intracellular ROS levels, and silencing TfR1 effectively attenuated ROS accumulation (Figure 5D,E), further supporting the involvement of TfR1 in NDRV‐induced oxidative stress. Collectively, these findings show that TfR1 is upregulated during NDRV infection and promotes macrophage ferroptosis by facilitating iron influx and ROS production. Thus, TfR1 represents a key molecular mediator in NDRV‐induced ferroptosis.

Figure 5NDRV induces ferroptosis in HD11 cells by upregulating the expression of TfR1. (A) HD11 cells were infected with NDRV for 24 and 48 h, followed by western blot analysis to detect protein expression levels of iron metabolism‐related genes. (B) Total RNA was extracted from cells under the same conditions, and mRNA expression changes of iron metabolism‐related genes were assessed by qPCR. (C) HD11 cells transfected with siRNA‐TfR1 were infected with NDRV, and intracellular Fe^2+^ levels were measured using FerroOrange. (D) In parallel experiments, the intracellular ROS levels in the treated cells were detected using the DCFH‐DA probe. (E) The effect of TfR1 knockdown on NDRV‐induced ROS accumulation was quantified by measuring the fluorescent signal area using ImageJ software. HD11 cells transfected with siRNA‐Hmox1 were infected with NDRV, and the expression levels of Hmox1, TfR1, and PTGS2 proteins were examined by western blot. Lipid peroxidation levels in siRNA‐Hmox1‐transfected HD11 cells following NDRV infection were determined using MDA assay kit. Significance levels are indicated as follows:  ^∗^ *p* < 0.05,  ^∗∗^ *p* < 0.01.

**Figure:**
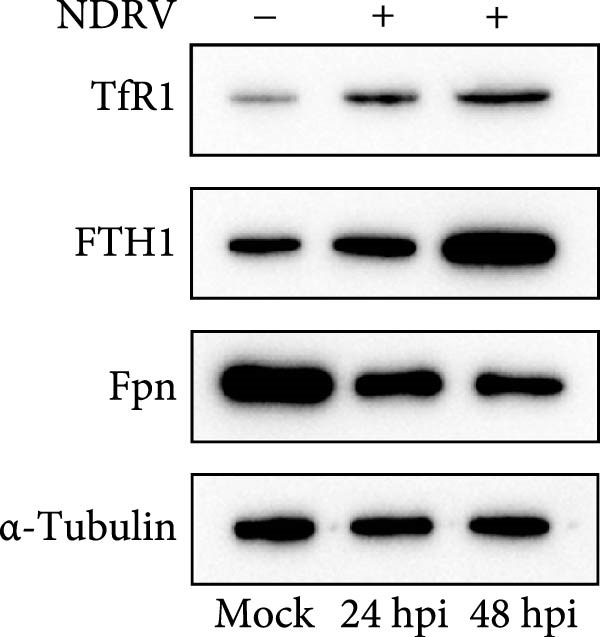
(A)

**Figure:**
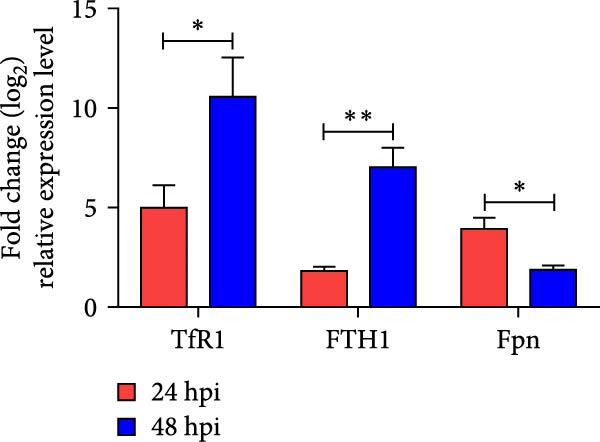
(B)

**Figure:**
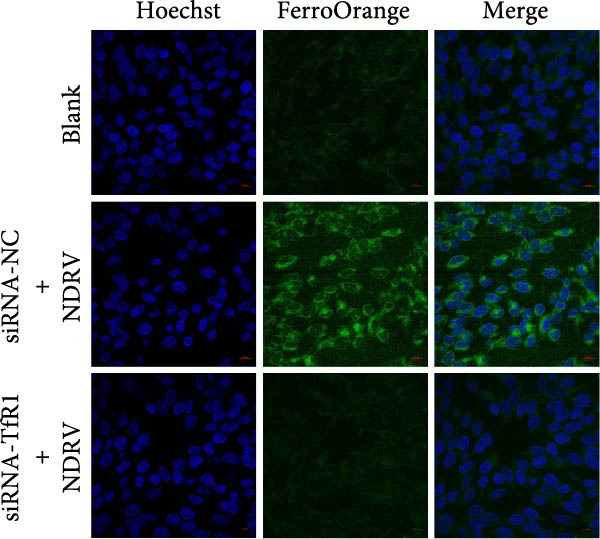
(C)

**Figure:**
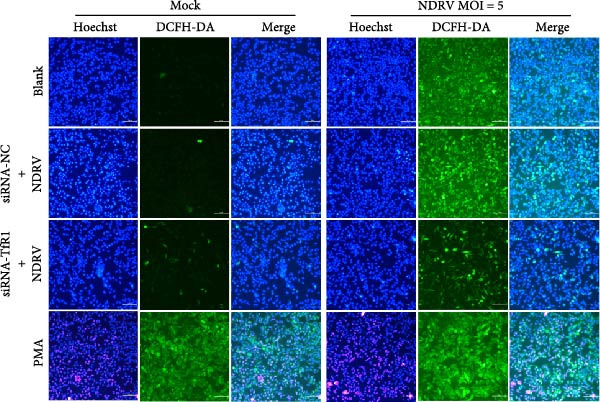
(D)

**Figure:**
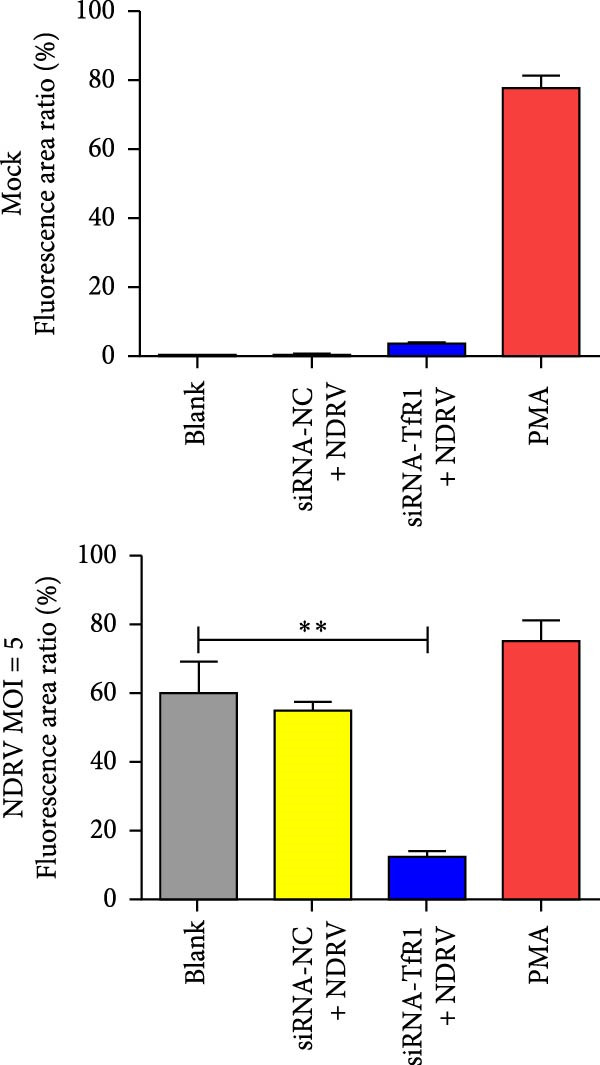
(E)

## 4. Discussion

The intricate interplay between viruses and host cells is pivotal in viral pathogenesis and the host immune response. Our previous study demonstrated that NDRV induces ferroptosis during splenic necrosis in ducks [17]. In the present study, we provide, for the first time, direct evidence that NDRV triggers ferroptosis in macrophages. Furthermore, we show that NDRV hijacks cellular iron metabolism to promote intracellular iron accumulation, thereby inducing ferroptosis in HD11 cells, with TfR1 identified as a key regulator in this process. These findings reveal the molecular mechanism by which NDRV causes host cell damage through disruption of iron homeostasis, providing a critical theoretical basis for elucidating the pathogenesis of NDRV‐induced splenic necrosis in ducks.

NDRV infection causes severe splenic necrosis in ducks, leading to immunosuppression, increased susceptibility to secondary infections, and vaccine failure [2]. However, the mechanisms of NDRV‐induced spleen injury and its capacity to evade host immune clearance remain unclear. ARV exhibits macrophage tropism [23], as supported by studies on orthoreoviruses of waterfowl origin [24, 25]. Previous research has established that splenic macrophages are the primary target cells of NDRV [7]. This study confirms that NDRV induces ferroptosis in HD11 cells by hijacking iron metabolism and promoting intracellular iron accumulation. Additionally, we show that the NDRV σA protein suppresses nuclear translocation of STAT1 and STAT2, thereby inhibiting interferon (IFN) signaling pathways. Although NDRV infection triggers IFN production in host cells, it exerts a significant inhibitory effect on IFN‐I‐mediated antiviral responses [17]. Notably, DRV has evolved novel pathogenic phenotypes over time. Li et al.[26] reported that the N‐DRV‐LY20 strain causes swelling of duck limb joints, along with locomotor and growth impairments. Sequence analysis indicates that the N‐DRV‐LY20 strain is phylogenetically closest to the previously reported NDRV‐XT18 strain [26]. MDRV infection in DF‐1 cells increases LC3‐II levels and reduces phosphorylated mTOR, indicating the induction of autophagy [27]. Further evidence indicates that both the σA and σNS proteins colocalize with LC3‐II, suggesting roles in autophagy and viral replication [28]. Therefore, the molecular basis for the diverse pathogenic phenotypes among different DRV strains, as well as their potential to induce alternative forms of cell death, warrants further investigation.

Ferroptosis, an iron‐dependent form of regulated cell death, is extensively implicated in the pathophysiological processes of various diseases. Numerous studies have shown that livestock and poultry pathogens can induce ferroptosis and exacerbate disease severity. For instance, IV [29], NDV [14], pseudorabies virus [30], African swine fever virus (ASFV) [31], porcine reproductive and respiratory syndrome virus (PRRSV) [32], porcine epidemic diarrhea virus (PEDV) [33], and *Staphylococcus aureus* [34] have all been shown to induce ferroptosis and contribute to disease progression. ASFV can induce oxidative damage and suppress IFN‐β production. Specifically, ASFV promotes ROS accumulation and lipid peroxidation through NCOA4‐mediated ferroautophagy, and the induction of STING carbonylation compromises cGAS‐STING pathway‐mediated antiviral immunity and suppresses IFN‐β production. Pharmacological inhibition of ferroptosis enhances the transcription of IFN‐β and ISGs [31]. Ferroptosis triggered by pathogen infection often leads to severe necrotizing inflammation. The ferroptosis pathway is significantly activated during swine IV (SIV) infection, and suppression of ferroptosis effectively inhibits viral replication and the associated inflammatory response [29]. Beyond infectious diseases, ferroptosis also plays a role in metabolic disorders and abiotic stress‐induced injuries. Aflatoxin B1 (AFB1) induces ferroptosis in duck kidney cells by causing mitochondrial oxidative stress and ferritinophagy [35].

Macrophages represent a critical component of systemic iron metabolism. In 2017, it was first reported that iron accumulation could induce ferroptosis in macrophages, and treatment with Fer‐1 was shown to mitigate associated liver injury [19]. Beyond iron accumulation exacerbating macrophage ferroptosis and tissue damage in RA [20], studies on atherosclerotic stress injury have demonstrated that macrophage P2Y12 receptors regulate iron metabolism via the NF‐κB/Hepcidin axis, promoting necrotic plaque formation [36]. Ferroptosis also represents a major mechanism underlying tissue necrosis during *Mycobacterium tuberculosis* (Mtb) infection. In infected macrophages, Mtb increases intracellular Fe^2+^, mitochondrial superoxide, and lipid peroxides—effects that can be reversed by Fer‐1. Fer‐1 treatment reduces bacterial load and improves lung injury [37]. Conversely, macrophages can resist pathogens by modulating ferroptosis. Early in bacterial infection, they trigger ferroptosis‐like bacterial killing through rapid rises in Fe^2+^ and lipid peroxidation. Supplementing macrophages with Fe^2+^ or iron‐based nanomaterials inhibits *Salmonella* and *Staphylococcus aureus* infection and reduces tissue damage [38]. Thus, understanding the interplay between iron metabolism and ferroptosis in macrophages is crucial for elucidating their role in pathological injury.

Targeting ferroptosis represents a promising strategy for the prevention and control of livestock and poultry diseases. On one hand, the induction of ferroptosis can suppress pathogen replication. Erastin significantly inhibits PEDV replication in Vero cells by upregulating Nrf2, ACSL4, and GPx4 expression, which is negatively correlated with PEDV infection, indicating that ferroptosis regulation mediates this antiviral effect [32]. On the other hand, inhibiting host cell ferroptosis can reduce tissue damage during infection. Iron accumulation acts as a defense mechanism against grass carp reovirus (GCRV) infection. GCRV suppresses viral replication and enhances cell survival by inducing oxidative stress through an increase in the LIP. The administration of FAC markedly reduced viral load and improved cell viability, indicating that iron supplementation may serve as a potential adjuvant therapy in the treatment of viral infections [39]. Therefore, further investigation into the role of ferroptosis in livestock and poultry pathogen infections is warranted to evaluate its potential as a therapeutic target.

TfR1 is a critical receptor mediating cellular iron uptake, and its upregulation enhances cellular susceptibility to ferroptosis. Accumulating evidence underscores the pivotal role of TfR1 in regulating ferroptosis. Hepatitis B virus (HBV) suppresses iron influx by targeting TfR1, thereby modulating host cell ferroptosis [40]. In D‐galactose‐induced aging rats, increased expression of iron regulatory protein 2 (IRP‐2) upregulates TfR1, promoting Fe^2+^ influx and inducing ferroptosis in auditory cortex neurons [41]. In diabetic cardiomyopathy (DCM), lysine acetyltransferase 2a (Kat2a) triggers ferroptosis by enhancing histone acetylation at the promoter regions of TfR1 and Hmox1, unveiling a previously unrecognized role of the Kat2a/Hmox1/TfR1 signaling axis [42]. This study is the first to show that NDRV infection upregulates TfR1 in HD11 cells, leading to iron accumulation and ferroptosis, thereby establishing a foundation for further investigation into the molecular interplay between NDRV proteins and TfR1.

In conclusion, this study demonstrates for the first time that NDRV induces ferroptosis in HD11 cells by disrupting iron metabolism and promoting intracellular iron accumulation, thereby providing a critical experimental foundation for understanding the mechanisms of NDRV infection. However, the specific signaling pathways through which NDRV regulates iron homeostasis and ferroptosis, the direct interactions between NDRV proteins and host iron‐ regulatory molecules, and the actual contribution of ferroptosis to viral pathogenesis in vivo remain incompletely characterized. Further research is needed to fully elucidate these mechanisms, which will not only enhance our understanding of NDRV pathogenesis but may also uncover novel insights and potential therapeutic targets for the prevention and control of NDRV and related viral infections.

## Disclosure

All the authors have read and approved the final version of the manuscript.

## Conflicts of Interest

The authors declare no conflicts of interest.

## Author Contributions

Conceived and designed the experiments: Hongzhi Wang, Yi Tang, and Rendong Fang. Conducted the experiments: Hongzhi Wang, Di Lei, Chenchen Jiang, and Boyi Xu. Analyzed the data: Hongzhi Wang, Di Lei, Chenchen Jiang, and Boyi Xu. Contributed reagents, materials, and analysis tools: Yi Tang and Rendong Fang. Wrote the paper: Hongzhi Wang, Di Lei, and Rendong Fang. Hongzhi Wang and Di Lei contributed equally to this work.

## Funding

This study was funded by the Chongqing Technology Innovation and Application Development Project (Grant CSTB2025TIAD‐GPX0004), the Fundamental Research Funds for Central University (Grants SWU‐XJPY202305 and SWU‐KQ22051), the Chongqing Modern Agricultural Industry Technology System (Grant CQMAITS202512), and the National Modern Agricultural Industry Technology System (Grant CARS‐42‐19).

## Data Availability

The data are available upon request from the author, Hongzhi Wang (whz20220904@swu.edu.cn), upon reasonable request.
